# Associations between abundances of free‐roaming gamebirds and common buzzards *Buteo buteo* are not driven by consumption of gamebirds in the buzzard breeding season

**DOI:** 10.1002/ece3.8877

**Published:** 2022-05-03

**Authors:** George J. F. Swan, Stuart Bearhop, Stephen M. Redpath, Matthew J. Silk, Daniel Padfield, Cecily E. D. Goodwin, Robbie A. McDonald

**Affiliations:** ^1^ 91046 Environment and Sustainability Institute University of Exeter Penryn UK; ^2^ Instituto de Conservación, Biodiversidad y Territorio Facultad de Ciencias Forestales y Recursos Naturales Universidad Austral de Chile Valdivia Chile; ^3^ 91046 Centre for Ecology and Conservation University of Exeter Penryn UK; ^4^ Institute of Biological and Environmental Sciences University of Aberdeen Aberdeen UK; ^5^ Present address: UK Centre for Ecology and Hydrology Wallingford UK

**Keywords:** buzzard, game management, pheasant, predation, raptors, rear and release

## Abstract

Releasing gamebirds in large numbers for sport shooting may directly or indirectly influence the abundance, distribution and population dynamics of native wildlife. The abundances of generalist predators have been positively associated with the abundance of gamebirds. These relationships have implications for prey populations, with the potential for indirect impacts of gamebird releases on wider biodiversity. To understand the basis of these associations, we investigated variation in territory size, prey provisioning to chicks, and breeding success of common buzzards *Buteo buteo*, and associations with variation in the abundances of free‐roaming gamebirds, primarily pheasants *Phasianus colchicus*, and of rabbits *Oryctolagus cuniculus* and field voles *Microtus agrestis*, as important prey for buzzards. The relative abundance of gamebirds, but not those of rabbits or voles, was weakly but positively correlated with our index of buzzard territory size. Gamebirds were rarely brought to the nest. Rabbits and voles, and not gamebirds, were provisioned to chicks in proportion to their relative abundance. The number of buzzard chicks increased with provisioning rates of rabbits, in terms of both provisioning frequency and biomass, but not with provisioning rates for gamebirds or voles. Associations between the abundances of buzzards and gamebirds may not be a consequence of the greater availability of gamebirds as prey during the buzzard breeding season. Instead, the association may arise either from habitat or predator management leading to higher densities of alternative prey (in this instance, rabbits), or from greater availability of gamebirds as prey or carrion during the autumn and winter shooting season. The interactions between gamebird releases and associated practices of predator control and shooting itself require better understanding to more effectively intervene in any one aspect of this complex social‐ecological system.

## Graphical abstract

Releasing gamebirds for sport shooting may influence the abundance, distribution, and population dynamics of native wildlife. Positive associations between the abundances of buzzards and pheasants may not be a consequence of the greater availability of pheasants as prey during the buzzard breeding season. Instead, the association may arise either from habitat or predator management leading to higher densities of alternative prey (in this instance, rabbits) or from the greater availability of gamebirds as prey or carrion during the autumn and winter shooting seasons.

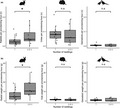

## 1 INTRODUCTION

In Europe, hunting gamebirds commonly involves the release of captive‐bred birds, alongside management of habitats and predator populations (Martinez et al., 2002; Park et al., 2008). Releases of reared gamebirds can be substantial. Each year, 43 million pheasants *Phasianus colchicus* and 9 million partridges (predominantly red‐legged partridge *Alectoris rufa*) are estimated to be reared and released in the United Kingdom (Madden, 2021), and one in twelve English woodlands contains a pheasant release pen (Sage et al., 2005). The scale of gamebird releases and the spatial extent of land managed for hunting released birds has prompted interest in their ecological effects (Mason et al., 2020; Mustin et al., 2018; Sage et al., 2020). One concern is that the increased prey densities associated with gamebird shooting might also encourage higher densities of generalist predators, leading to increased predation pressure on other wildlife, as well as upon the gamebirds themselves (Lees et al., 2013; Swan et al., 2020).

In a broad‐scale analysis using three extensive datasets, Pringle et al. (2019) analyzed spatial and temporal variation in the abundances of reared and released, and free‐roaming, gamebirds (i.e., pheasants and red‐legged partridges) and of generalist avian predators (i.e., common buzzard *Buteo buteo*, carrion crow *Corvus corone*, and jay *Garrulus glandarius*). They identified a series of predominantly positive associations, and suggested that gamebird releases, and consequently increased free‐roaming gamebird populations, have a significant positive effect on both the abundance and inter‐annual population growth rates of some of these generalist predators. On this basis, they identified the potential for increased predator populations, subsidized by gamebird releases, to have indirect effects upon wider biodiversity.

The identification of relationships between gamebird releases and predator abundances represents an important advance. However, the ecological mechanisms that might underlie these relationships have not been explored to the same extent (Ludwig et al., 2020). In the meantime, restrictions upon gamebird releases were suggested by Pringle et al. (2019) as a potential conservation tool for wild bird populations. Regulation of this, or other practices associated with game‐shooting, would be most effective if targeted at the salient mechanisms and drivers of any undesired outcomes. Management of land for gamebird shooting might influence predator densities and breeding success either directly, by fostering high densities of gamebird prey, or indirectly, by encouraging high densities of alternative, non‐game, prey species, due to food provision, predator control or habitat management (Madden & Sage, 2020).

To disentangle these possibilities, and to identify whether and where regulation might be effective, requires more focused study of the mechanisms underpinning observed relationships and of predator responses to variation in abundances of gamebirds and other potential prey (Park et al., 2008). Because of the contentious nature of game management practices, including gamebird releases, and their potential effects on non‐game wildlife, further investigation is needed to uncover the drivers of any relationships between gamebird abundances and predator responses. For these purposes, local‐scale analyses based on direct observations on land managed for gamebird shooting is better suited to exploring potential mechanisms, and identifying any issues that might mask or bias underlying relationships.

In specific relation to buzzards, Pringle et al. (2019) identified a positive, landscape‐scale association between the number of free‐roaming pheasants and buzzard abundance during the breeding season. Although this suggests that buzzard populations might be supported by gamebirds as a food resource, their analysis was necessarily conducted at a very large scale and relied upon assumptions that the scale of releases are similar among years, and that point source releases are spatially correlated at the scale of predator population assessments. Their analyses were, again necessarily, correlative and so could not establish a cause‐and‐effect relationship. Indeed, their study tested, but found no significant relationship between, buzzard population growth rates and numbers of pheasants, as might have been expected, if gamebirds were important prey.

The common buzzard is a dietary generalist and is able to utilize a wide variety of prey and food sources (Francksen et al., 2016; Graham et al., 1995; Swan, Bearhop, et al., 2020). This allows buzzards to respond to increases in food availability both at a population level, through increased territory size and productivity (numerical response), and at an individual level, by increasing how often they consume the item (functional response; Francksen et al., 2017). The annual release of young reared pheasants (poults) occurs during late June and July, by which time most buzzard nestlings are well‐developed and mortality rates are low (Hardey et al., 2013; Kenward et al., 2001; Rooney & Montgomery, 2013). Although poult releases may play a role in post‐fledging survival and eventual recruitment, it appears unlikely that they will directly influence the density of buzzard nests or the number of nestlings in them. This research, therefore, focuses on those 'free‐roaming' gamebirds that have been released in previous seasons or have hatched in the wild.

We examined how buzzard ecology during the breeding season is influenced by local abundances of free‐roaming gamebirds, and by their provisioning rates by adult buzzards to young in nests, on, and around, lowland pheasant shooting estates. We then made the same comparisons with two other important prey species for buzzards: rabbits *Oryctolagus cuniculus* and field voles *Microtus agrestis*. Specifically, we tested whether buzzards responded to higher abundances of the three prey species in three ways: (i) increase in territory size, (ii) increase in provisioning rates to nestlings, and (iii) increased chick numbers.

## 2 MATERIALS AND METHODS

### 2.1 Study sites

Fieldwork was undertaken from April to August 2015 on three study sites in Cornwall, UK (50°21’N, 4°49’W). Site A (9 km^2^) was 6 km from Site C (9 km^2^) and both were approximately 25 km from Site B (11 km^2^). Over all sites, the habitat in buzzard territories, according to the UK Land Cover Map at 25 × 25 m resolution, was comprised of improved grassland (39%), arable and horticulture (35%), broadleaf woodland (23%), coniferous woodland (2%), and suburban (1%). The habitat compositions of sites A and C were broadly comparable, although site B contained more broadleaf woodland (40.2% compared to 12.6% and 16.3% of A and C, respectively) and less arable or horticultural land (11.8% compared with 46.6% and 44.8% of A and C, respectively; Appendix Table S1). All sites were centered around private shooting estates, where management for gamebird shooting included the annual release of >10,000 pheasant poults during late June and July. The numbers of birds released in the previous year were not available, for reasons of confidentiality, although the gamekeepers self‐reported their releases on one of the sites (A) as larger than average, and the other two sites (B and C) as average. Other multi‐site studies in southern England report release densities in pens of 1489–2251 birds/ha as typical (Neumann et al., 2015; Sage, Ludolf, et al., 2005). Gamekeepers on all estates focused predator control on reducing red fox *Vulpes vulpes* density (estimated at a county level at 2.6 and 3.6 foxes/km^2^; Parrott et al., 2012 ); however, mustelids (stoat *Mustela erminea* and weasel *M*. *nivalis*) and corvids (mainly carrion crow *Corvus corone* and magpie *Pica pica*) were also targeted with effort that varied between sites.

### 2.2 Territory mapping

Buzzard breeding territories were mapped by locating active buzzard nests through systematic searches of all woodland, tall hedgerows, and lone trees during April and May 2015. A nest was considered active upon observation of an adult bird leaving the nest. Once all nests had been located, the nearest neighbor distance (NND) to another nest was calculated using QGIS as an approximation of territory size (Prytherch, 2013; Swann & Etheridge, 1995). Although not a measure of density in itself, there is strong evidence that NND and breeding density are tightly correlated in buzzard populations (Prytherch, 2013). Half of the mean NND of all nests was then used as the “core territory” radius for all nests. Active nests were accessed once during the early provisioning period (~7 days after hatching) to install nest cameras, and then again when nestlings were between 18 and 25 days old for the purposes of fitting British Trust for Ornithology (BTO) leg rings. All research received prior ethical approval from the University of Exeter Animal Welfare and Ethical Review Board and was conducted by trained and experienced personnel under licences from the BTO (CO/6164) and Natural England (2015‐7805‐SCI‐SCI).

### 2.3 Woodland cover

As habitat composition may influence the distribution and abundance of buzzards and their prey (Swan, 2011), the percentage of 'woodland cover' (broadleaf woodland and coniferous woodland) was obtained for each territory in QGIS using a UK Land Cover Map Vector at 25 × 25 m resolution for the year of data collection (2015).

### 2.4 Prey abundance

As buzzard pairs provision their young with prey hunted within established territories (Prytherch, 2013), the area within the core territory of each nest was used to sample prey abundance. Where core territories overlapped, boundaries were assigned using Thiessen polygons. In addition to free‐roaming gamebirds, the relative abundances of rabbits and field voles were quantified within each buzzard core territory (following Graham et al., 1995); as these species are known to be an important prey source from buzzards in the UK (Francksen et al., 2016). Measures of relative prey abundance for each of the three prey groups were recorded at 40 points (randomly assigned throughout the territory using QGIS) in all 37 territories (1480 points in total). This was achieved immediately after the nestlings had fledged (July–early August) to minimize disturbance to the nest and prevent premature fledging. Thus, there was an interval of 1–2 months between these two periods, creating some temporal disparity in our data.

For rabbits, an adaptation of the 'standing crop pellet count' (Fernandez‐de‐Simon et al., 2011) was followed whereby, rather than searching a 0.5 m^2^ quadrat for rabbit pellets at regularly spaced intervals along a transect, we searched a randomly placed 1 m^2^ quadrat at every sampling point in each territory. Brown hare *Lepus europaeus* droppings were distinguishable from rabbit droppings but were not counted as hares are only found in very low densities in Cornwall (Parrott et al., 2012 ) and rarely feature in buzzard diet (Swan, Bearhop, et al., 2020). The total number of quadrats per territory in which rabbit droppings were located was then used as a relative index of rabbit abundance. For voles, the top right corner (25 x 25 cm) of the same quadrat was examined for the presence or the absence of field vole signs, specifically grass clippings and droppings. Following Lambin et al. (2000), this area was then scored 0, 1 or 2 depending on the presence or deterioration of the clippings (0 = none, old = 1, fresh = 2). This score was then summed for each territory to create a field vole sign index.

To provide a measure of the relative abundance of free‐roaming gamebirds for each territory timed point counts conducted whereby at each point, all pheasants and red‐legged partridges observed within 100 m over 2 min were recorded. Point counts followed methods detailed in Selås et al. (2007), although we extended the maximum observation distance and reduced the observation time from the original methodology as our interest was in gamebirds rather than the whole bird community. Distances were recorded from where the birds were first seen (to account for disturbance) and were measured using a laser rangefinder (Rangemaster 1600, Leica Cameras). Recently released juvenile pheasants (observed only in proximity to release pens and identifiable from uniform plumage, clipped primaries and the absence of an associated adult bird) were excluded from this index. We used two, related measures of relative gamebird abundance from these surveys: First, the total number of points in each territory from which gamebirds were observed to be present (gamebird presence index) and second, the total number of gamebirds counted in the territory (gamebird total count index).

### 2.5 Provisioning rates and biomass at nests

Observations of provisioning of nestlings by adult buzzards were collected using motion‐activated remote cameras (CMOS 380 TVL, HandyKam, Cornwall) installed on accessible nests with nestlings present. Although this method can include its own biases, such as underestimating small or difficult to identify prey, recording of provisioning in this way represents the most accurate technique for determining food habits at raptor nests (García‐Salgado et al., 2015; Lewis et al., 2004). Dietary analysis from prey remains at the nest site was not used in this study as it is likely to overestimate the importance of gamebirds in buzzard diet (Francksen, Whittingham, & Baines, 2016; Swan, Bearhop, et al., 2020). Cameras were active during daylight hours (average first and last images were taken at 06:37am and 20:24pm, respectively) and, upon detecting movement, recorded up to 5 min of continuous video footage. Videos of prey deliveries were watched by a single observer (GS). Alongside provisioning frequency, biomass was estimated for each prey item to provide a more appropriate measure of relative importance in buzzard nestling diet. This was achieved following methods established in Swan, Bearhop, et al. (2020), whereby a mass was allocated to each item based on species, size, and proportion provisioned. For unidentified items, approximate size and time taken to consume the item were used to estimate mass (Swan, Bearhop, et al., 2020). Free‐roaming pheasants and those that were released that year were differentiated by the presence (released poults) or the absence (free‐roaming pheasants) of clipped primary feathers on one wing (Appendix Figure S1). This is a temporary procedure (clipped juvenile primaries are replaced by adult primaries at ~10 weeks old) undertaken by gamekeepers to deter initial dispersal. Although it was not possible to differentiate between field voles and bank voles *Myodes glareolus* in the video footage, analysis of regurgitated pellets suggests that, bank voles are comparatively rare in buzzard diet (Graham et al., 1995). Only nests with >25 h of camera observations were included in the analysis to limit potential bias arising from short observation periods.

### 2.6 Statistical analysis

To standardize the indices of prey relative abundance, values for each were scaled (by subtracting the mean and dividing by the standard deviation). They were then checked for collinearity using Spearman's rank correlation. To check for underlying variation in the relative abundance indices of prey among the three study sites, linear models were used with the prey abundance indices as the responses and site as a categorical explanatory variable.

To investigate whether there was a relationship between buzzard territory size and the relative abundances of their prey, we fitted a linear model with nearest neighbor distance, as an index of territory size, as the response (log‐transformed to meet assumptions of normality) and the relative abundance indices for rabbit, vole, and gamebirds (presence index) as explanatory variables. Site and % woodland cover were included as fixed effects to control for underlying spatial variation in density or predator management regimes (site) and available nest sites, habitat, and prey detectability (woodland cover). This analysis was then repeated, replacing the gamebird presence index with the total count index.

Using observations from camera footage, the provisioning rates for rabbits, voles, and gamebirds were calculated for each nest for both frequency and biomass (total observations for each prey group or total weight for each prey group / total number of hours the camera was running). To investigate whether buzzards utilize rabbits, voles and gamebirds in proportion to their relative abundance, three linear models were fitted with provisioning rate for each of the three prey groups as the response variable and the relative abundance for that prey as the explanatory variable. For the analysis of gamebird provisioning rates, separate analyses were run for the gamebird presence index and the total count index. Prior to inclusion, the provisioning rates were log‐transformed to meet model assumptions and as some measurements of provisioning rate were zero, a constant value of 0.001 was added to all observations.

We explored the influence of provisioning rate and biomass provisioned for the three prey groups on buzzard productivity in two separate analyses. Nests that had failed (an empty nest after an adult had been seen incubating) were not included in these analyses of provisioning rates as it was not possible to collect sufficient dietary data. First, variation in buzzard chick numbers was analyzed by fitting a logistic regression with the number of nestlings, categorized as 1 or >1 (nests containing two and three nestlings were grouped as only two nests had three nestlings), as a binomial response variable, with the provisioning rates (frequency per provisioning hour) of rabbit, vole, and pheasant, and site and woodland cover as fixed effects. As the three prey groups had different weights, we then conducted the same analysis using their biomass provisioning rates (grams per provisioning hour) as explanatory variables. The significance of terms in regression models was assessed by comparing the amount of variation explained by models with, and without, the term using ANOVA and F‐tests (for Gaussian response variables) and Chi‐square tests (for binomial response variables). Model checks included running models with and without influential data points (identified as having a Cook's distance >0.5). R version 4.0.2 (R Core Team, 2020) was used for all analyses.

## 3 RESULTS

A total of 37 active buzzard territories were located and mapped (11, 13 and 13 nests distributed across the three sites). The average nearest neighbor distance was 690 m (SD = 202 m, range = 485–1150 m, *n* = 36; one nest was excluded as it was on the periphery of a study site and NND was uncertain) and core territories were assumed to be within a 345 m radius from each nest. Thus, the likely areas of buzzard core territories were considerably larger than the ranges of their prey (Harris & Yalden, 2008; Hill & Robertson, 1988), supporting the independence of indices among territories. Of the 37 active territories, 27 (73%) still contained nestlings at the second nest check (at 18–25 days old).

### 3.1 Provisioning observations

Cameras were installed to sample prey provisioning at 24 of the 27 nests with nestlings, however, one of these was excluded as it recorded <25 h of observations and so was excluded from the analysis. The remaining 23 cameras recorded a total of 4279 ‘provisioning hours’ over 305 days. At each nest, cameras were active for an average of 13.3 days (SD = 6.1, range = 2–24) and encompassed an average of 186 ‘provisioning hours’ (SD = 97, range = 25–370). 1450 provisioning events were observed (mean number per nest = 63, SD = 35, range = 8–129) representing 94.3 kg of biomass (mean biomass per nest = 4.1 kg, SD = 2.1, range = 1.6–8.2; Table 1). Of the provisioned items, 82% were identified to the desired taxonomic level. Of 262 unidentified items, 247 were small items <50 g (Table 1), and so 93.7% of biomass comprised identified prey. The three focal prey groups (rabbits, voles, and gamebirds) comprised 43.4% (*n* = 629) of the observations and 60.5% (57.1 kg) of the total biomass (Table 1). Voles were the most frequently delivered prey item (*n* = 365, 25.2% frequency) and made up 6.9% of total biomass. Rabbits were the second most frequently delivered prey item (*n* = 195, 13.4% frequency), but were the most important for biomass (39.2% of total biomass). Gamebirds were recorded in 69 provisioning events (4.8% total frequency, 14.4% total biomass), of which, 1 (0.1% frequency, 0.4% biomass) was identified as a red‐legged partridge, 29 (2.0% frequency, 5.7% biomass) as free‐roaming pheasants and 39 (2.7% frequency, 8.3% biomass) were identified as released pheasants (reared that year). Of the free‐roaming pheasants, 9 were chicks (estimated to be <3 weeks old), 14 were juveniles (3–10 weeks old), and 7 were adult birds (>10 weeks old), based on size and plumage. The released pheasants were mainly observed toward the end of the provisioning observation period as annual releases occur during late June and July. As our research interest is in the effect of free‐roaming gamebirds on buzzard breeding ecology, we excluded released pheasants from the analysis, except where stated otherwise.

**TABLE 1:** Summary of frequency and biomass of prey items provided by adult common buzzards to chicks in 23 nests in Cornwall, UK. Prey items were observed on video footage from remote cameras. The three prey groups investigated in this study are shown in bold. Biomass was estimated for each prey item based on species, size and proportion provisioned

Prey category	Species	Frequency of occurrence		Total biomass	
		*N*	%*N*	g	%
Rabbits	*Oryctolagus cuniculus*	195	13.4	36,990	39.2
Voles	*Myodes glareolus / Microtus agrestis*	365	25.2	6541	6.9
Other mammals		216	14.9	14,494	15.4
Gamebirds	*Phasianus colchicus* (free‐roaming)	29	2.0	5418	5.7
	*Phasianus colchicus* (released)	39	2.7	7836	8.3
	*Alectoris rufa*	1	0.1	342	0.4
Other birds		111	7.7	9810	10.4
Amphibians		213	14.7	5935	6.3
Reptiles		8	0.6	462	0.5
Invertebrates		9	0.6	37	0.0
Fish		2	0.1	600	0.6
Unidentified	Total unidentified items	262	18.0	5925	6.3
	‐ Unidentified small mammals	109	7.5	1599	1.7
	‐ Small item (<50g)	138	9.5	2236	2.4
	‐ Medium item (50‐150g)	10	0.7	1040	1.1
	‐ Large item (>150g)	5	0.3	1050	1.1
Total		1450	100	94,390	100
Total identified		1188	81.9	88,466	93.7
Total in 3 focal prey groups		629	43.4	57,128	60.5

### 3.2 Prey indices

Relative indices of abundance were collected for rabbits (mean = 4.6 ± S.D. 4.9, range 0–18), field voles (11.5 ± 4.5, range 2–22), and gamebirds (Presence: 4.6 ± 4.8, range 0–23; Total Count: 8.9 ± 11.2, range 0–53; frequency histograms are presented in Appendix Figure S2). There was a significant positive correlation between the relative abundance index for rabbits and both free‐roaming gamebird indices (Spearman's rank correlation; Presence: *r_s_* = 0.349, *p* = .034; Total Count: *r_s_* = 0.354, *p* = .032), a negative but statistically non‐significant correlation between rabbits and voles (*r_s_* = −0.310, *p* = .061), and no correlation between voles and both free‐roaming gamebird indices (Presence: *r_s_* = 0.065, *p* = .762; Total Count: *r_s_* = 0.006, *p* = .971). Of the three prey groups (including both gamebird indices), only the relative abundance indices of rabbits varied significantly among sites (*F*_2,34_ = 20.49, *p* < .001).

**FIGURE 1:**
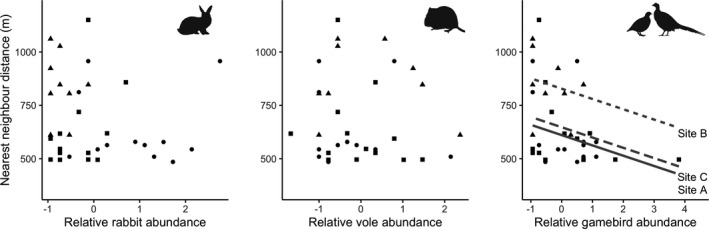
Relationships between the nearest neighbor distance of 36 active common buzzard nests in Cornwall, UK, and the indices of relative abundance for rabbits (*left*), field voles (*middle*) and free‐roaming gamebirds (*right*) prey. Lines indicate statistically significant relationships between nearest neighbor distance and indices of relative abundance of gamebirds, and variation among sites. Symbols indicate study sites: A (circles), B (triangles), and C (squares)

The nearest neighbor distance of 36 buzzard territories was negatively, albeit weakly, affected by the presence/absence index of gamebird abundance (*F*_1,30_ = 4.20, *p* = .049; Figure 1) with NND decreasing by 8% (95% CI: 0–16%) for every increase of 1 in the index. In the similar analysis using the total count index of gamebird abundance there was a negative, but statistically non‐significant correlation (*F*_1,30_ = 3.87, *p* = .059), although effect sizes were similar. No significant effects were observed for the rabbit index (*F*_1,30_ = 0.35, *p* = .557), vole index (*F*_1,30_ = 0.13, *p* = .722; Figure 1), percentage of woodland in territory (*F*_1,30_ = 0.23, *p* = .632), or site (*F*_2,31_ = 2.66, *p* = .087).

**FIGURE 2:**
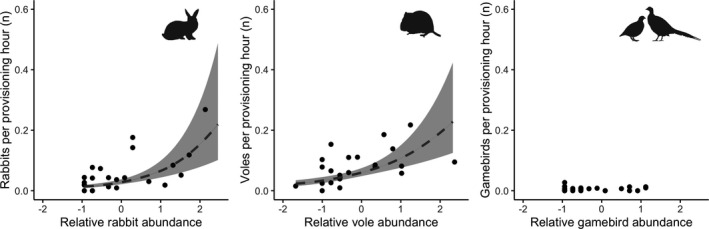
Relationships between prey provisioning rates and abundance indices for rabbit (*left*), vole (*middle*) and free‐roaming gamebirds (*right*) in 23 common buzzard territories in Cornwall, UK. Provisioning rates are numbers of items provisioned per hour of nest camera footage. The dashed line indicates a statistically significant relationship between the provisioning rate for rabbits and rabbit relative abundance and the provisioning rate for voles and vole relative abundance. The area shaded grey denotes the standard error of the predictions

Prey provisioning rates (returns per provisioning hour) in territories with nest cameras (*n* = 23) were significantly and positively related to abundance indices for rabbits (*r^2^* = 0.24, *F*_1,21_ = 7.86, *p* = .011; Figure 2) and voles (*r^2^* = 0.19, *F*_1,21_ = 6.25, *p* = .021) with the rabbit provisioning rate increasing by 131% (95% CI: 24–329%) and the vole provisioning rate increasing by 78% (95% CI: 10–189%) for every increase of 1 in their respective abundance indices. No significant relationship was observed in the provisioning rates and the gamebird presence index (*r^2^* = −0.05, NB adjustment of r^2^ for small sample size can create a negative r^2^ value; *F*_1,21_ = 0.00, *p* = .993; Figure 2) or the gamebird total count index (*r^2^* = −0.04; *F*_1,21_ = 149, *p* = .703).

**FIGURE 3:**
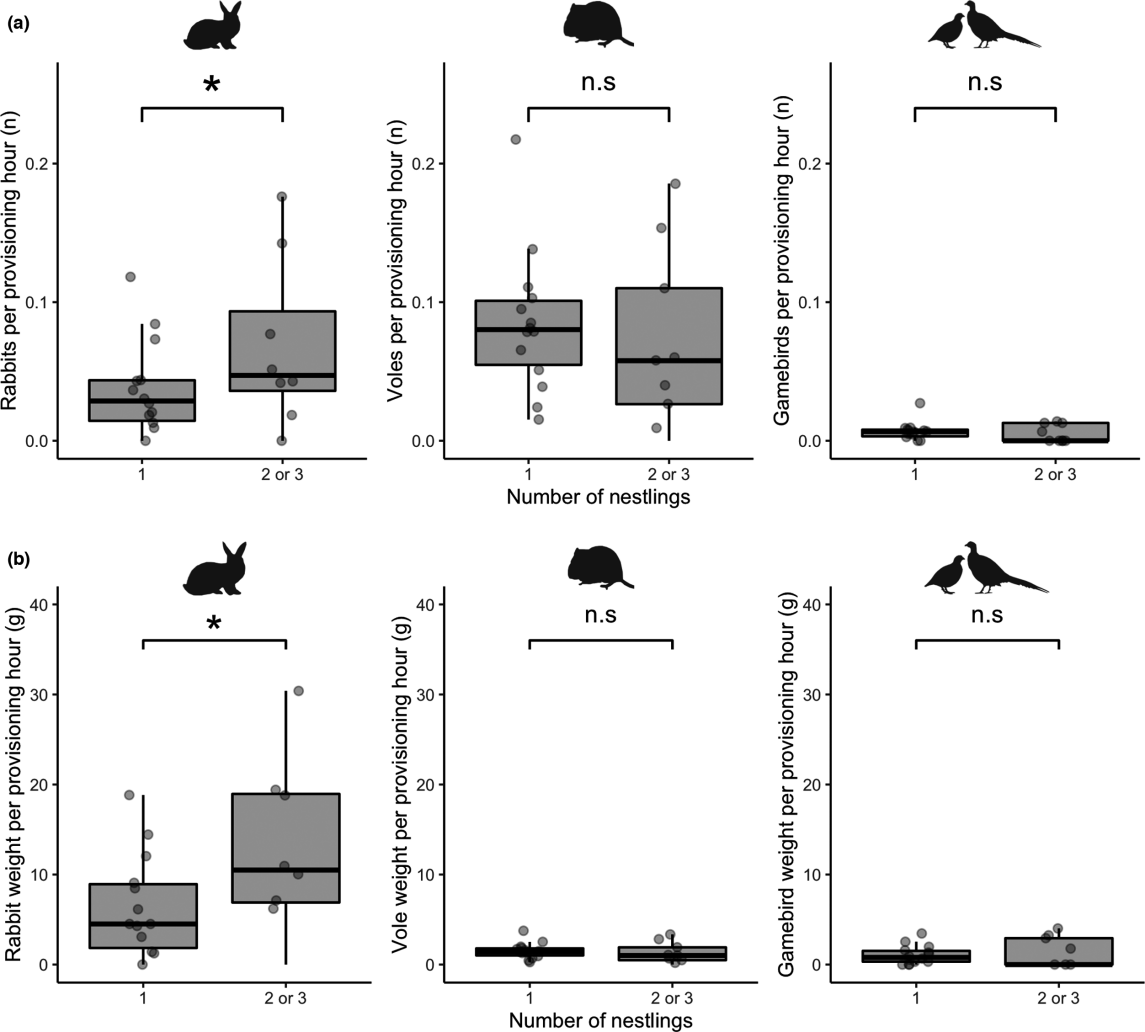
Variation in provisioning rates (a) items per hour and (b) biomass per hour, of rabbits (*left*), voles (*middle*) and free‐roaming gamebirds (*right*) at 23 common buzzard nests in Cornwall, UK, by the number of nestlings per nest. Boxplots indicate the median and interquartile range, whiskers indicate largest/smallest observation ±1.5× the interquartile range. Stars denote the significant effects of rabbit provisioning rates on nestling number

**FIGURE 4:**
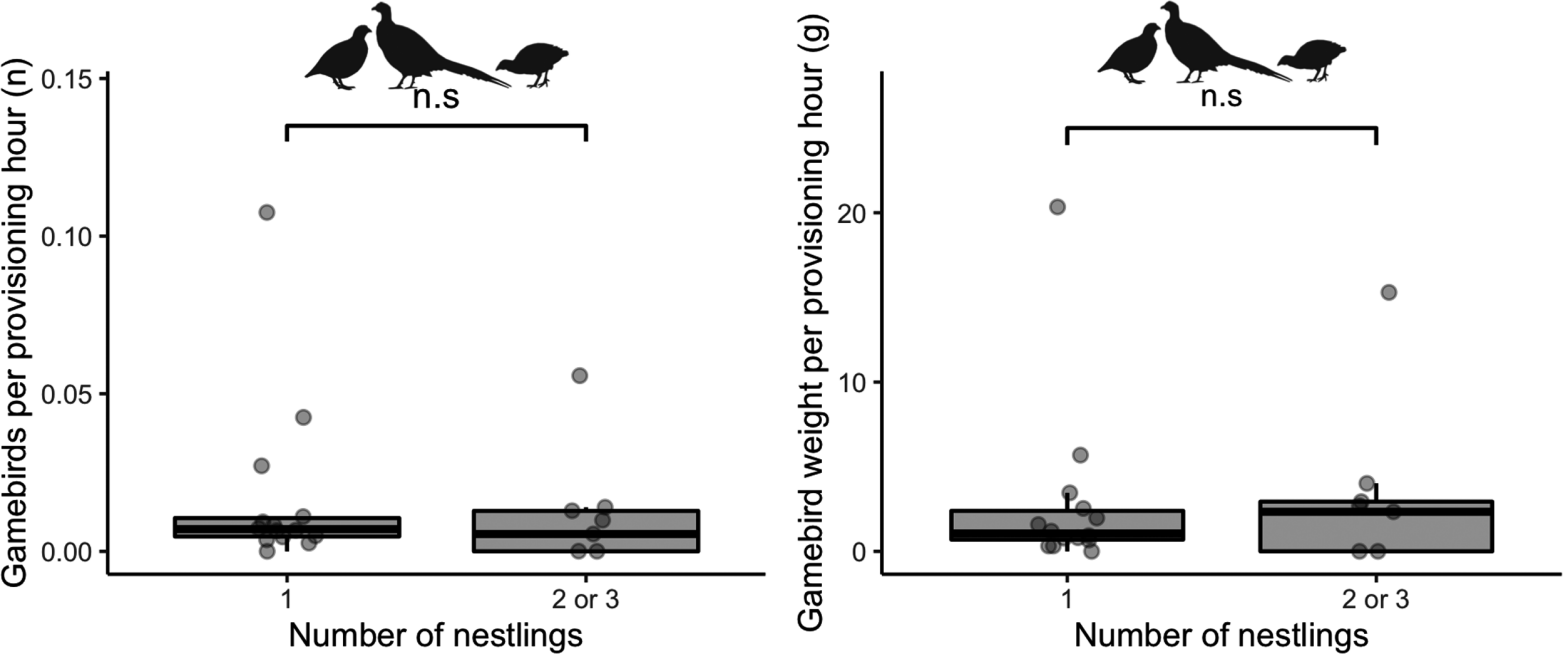
Variation in provisioning rates, items per hour (*left*) and biomass per hour (*right*), of all gamebirds (free‐roaming gamebirds and released pheasant poults combined) at 23 common buzzard nests in Cornwall, UK, by the number of nestlings per nest. Boxplots indicate the median and interquartile range, whiskers indicate largest/smallest observation ±1.5× the interquartile range

At the time of BTO leg‐ringing, the territories with nest cameras installed (*n* = 23) contained 14 nests with 1 nestling, 7 nests with 2 nestlings, and 2 nests with 3 nestlings. The presence of more than one nestling was significantly and positively associated with the provisioning rate per hour for rabbits ($X_{1}^{2}$ = 4.32, *p* = .038; Figure 3a) with a 25% increase in the odds of a nest having more than one chick for every 0.01 increase in rabbits per hour (OR 1.25, 97.5% CI: 1.01–1.76). No statistically significant effect was observed for voles ($X_{1}^{2}$ = 0.01, *p* = .903), free‐roaming gamebirds ($X_{1}^{2}$ = 0.00, *p* = .972; Figure 3a), woodland cover ($X_{1}^{2}$ = 0.58, *p* = .440) or site ($X_{2}^{2}$ = 0.79, *p* = .673). We also observed that the presence of more than one nestling was significantly and positively associated with the provisioning biomass per hour for rabbits ($X_{1}^{2}$ = 6.29, *p* = .012; Figure 3b), with a 17% increase in the odds of a nest having more than one chick for every 1 gram increase in rabbit biomass per hour (OR 1.17, 97.5% CI: 1.02–1.49). Again, no significant association was observed for voles ($X_{1}^{2}$ = 0.17, *p* = .677), free‐roaming gamebirds ($X_{1}^{2}$ = 0.91, *p* = .339; Figure 3b), woodland cover ($X_{1}^{2}$ = 0.31, *p* = .575), or site ($X_{2}^{2}$ = 0.48, *p* = .786). Finally, when the 39 observations of released pheasant poults (otherwise excluded from the analysis) were included with the 30 free‐roaming gamebirds, it did not alter the lack of an effect of gamebird provisioning rate per hour ($X_{1}^{2}$ = 0.00, *p* = .985) or gamebird provisioning biomass per hour ($X_{1}^{2}$ = 0.43, *p* = .513) on nestling numbers (Figure 4).

## 4 DISCUSSION

We have evaluated associations between buzzard prey provisioning rates, territory size and productivity, and the abundances of gamebirds, rabbits and field voles on shooting estates. We first found positive, albeit relatively weak, associations between measures of buzzard abundance, in this case nearest neighbor distance, and gamebird abundance. This pattern at a local scale is consistent with the positive relationship between pheasant and buzzard abundances at a national scale, identified by Pringle et al. (2019). However, our wider analysis included relative abundance, not only of gamebirds, but also of other important prey species, and quantified their provisioning rates from nest cameras. By so doing, we provide evidence that gamebirds are not provisioned in proportion to their abundance and that provisioning of gamebirds does not affect buzzard productivity. Instead, our results suggest that it is rabbits that are the most important prey for breeding buzzards, as pairs in territories with relatively high rabbit abundance provisioned them to their nests at a higher rate and, as the provisioning rate of rabbits increased, raised more chicks.

In contrast to the important contribution of rabbits to buzzards during the breeding season, we found little evidence that gamebirds were an important food resource at this time, as they were rarely brought to the nest, were not provisioned in proportion to their abundance, and pairs provisioning more gamebirds did not have more nestlings. Although these findings concern only the period when nestlings are being provisioned, they may explain why Pringle et al. (2019) did not observe a statistically significant association between pheasant abundance and buzzard population growth rates. They also partly answer concerns that artificially high gamebird densities provide a food resource that might specifically elevate breeding success in local buzzard populations (Mason et al., 2020; Swan, Redpath, et al., 2020).

The relationship between free‐roaming gamebird abundance and nearest neighbor distances between buzzard nests is perhaps surprising, considering their low relative contribution to buzzard nestling diet. There was a significant positive correlation between the relative abundances of rabbits and gamebirds at the territory level and so it is possible that habitat on land managed for shooting may also provide buzzards with greater densities of other prey sources. Specifically, shooting estates are more likely to maintain hedgerows and woodland belts (Oldfield et al., 2003) or plant game crops (Sage et al., 2005). A recent meta‐analysis found that habitat management for gamebirds in agricultural areas had largely positive benefits on non‐game wildlife (Mustin et al., 2018). Rabbits tend also to occur at higher densities on land where mammalian predators, such as foxes, stoats and weasels, are removed (Beja et al., 2008; Trout & Tittensor, 1989) and such legal control of mammalian predators, as commonly practised by gamekeepers (McDonald & Harris, 1999; Swan, Bearhop, et al., 2020), might enable the “competitive release” (Trewby et al., 2008) of buzzard populations, allowing them to reach unusually high densities.

An alternative or complementary explanation for the, albeit weak, relationship between our indices of buzzard territory size and pheasant abundance could be that gamebirds are of greater dietary importance in late winter or early spring, that is after fledging of juveniles and the period when adults select nest sites and define territories (Prytherch, 2013; Tubbs, 1974). It is possible that buzzards increase their consumption of gamebirds over this period, and gamekeeper records of raptor predation on red‐legged and gray partridges *Perdix perdix* peak between February and May (Watson et al., 2007). It is also plausible that areas with more pheasant releases provide more carrion during the winter and early spring period, as a consequence of unrecovered shooting casualties (Taggart et al., 2020; Watson et al., 2007) and vehicle collisions (Madden & Perkins, 2017). For example, in their study of gray partridge survival, Watson et al. (2007) estimate 10% of the birds that died following shooting were not recovered. Although such data are unavailable for the gamebirds in this study (pheasants and red‐legged partridges), this would represent a sizable biomass of carrion, potentially allowing buzzard pairs to maintain smaller territories. Indeed, a recent analysis of 187 UK buzzard livers found that lead concentrations increased substantially within years during the hunting season, providing strong evidence that buzzards are consuming gamebirds killed, or injured, in the course of shooting (Taggart et al., 2020). Further investigation into the determinants of buzzard recruitment and breeding densities will require data on both the diets of juvenile birds after fledging and the winter diets of adult territory holders, especially in areas managed for lowland gamebird shooting. This will necessitate methods that can be applied without the nest as a focal point, such as direct observations (Redpath et al., 2002), collecting pellets at roosts (Francksen, Whittingham, Ludwig, et al., 2016) or, if birds can be captured and their tissue sampled, dietary stable isotope analysis (Swan, Bearhop, et al., 2020).

Our results provide further confirmation of the importance of rabbits in both the diet and breeding success of buzzards (Rooney & Montgomery, 2013; Sim et al., 2001; Swann & Etheridge, 1995). Unlike previous studies (Graham et al., 1995), we did not observe a significant relationship between our indices of rabbit abundance and buzzard territory size. However, rabbits were the only prey species for which the abundance of which varied significantly among study sites and the inclusion of site in the model, in order to control for other potential sources of between‐site variation, may have made this relationship difficult to discern. Like others (Francksen et al., 2017; Ludwig et al., 2020), we observed that, although buzzards provisioned field voles in relation to their abundance, voles did not influence breeding success. Despite voles being the most frequently provisioned prey by number, it is likely that estimates of provisioning (both rate and biomass per hour) used in this study are underestimates, as smaller prey items, like small mammals, tend to be eaten more quickly and so are more difficult to identify on cameras (García‐Salgado et al., 2015; Swan, Bearhop, et al., 2020; Table 1). Instances when voles were recorded as “unidentified small prey” were assumed to occur randomly among territories, and therefore, the statistical findings of this study should remain valid. A further limitation of this study relates to the temporal disparity (1–2 months) between prey abundance sampling and the provisioning observations. The short interval between these two periods means changes in the relative abundance of prey are unlikely to have greatly affected the results. Indeed, temporal disparities of this size are not uncommon in raptor studies that compare prey abundance to nest‐based diet assessments or to breeding variables (e.g., Francksen et al., 2017; Redpath et al., 2002; Reif et al., 2004).

## 5 CONCLUSIONS

The ecological associations between predators and gamebird releases are the subject of interest due to concerns that such releases may subsidize higher predator densities. In this study, whilst buzzard territories were smaller in areas of relatively great gamebird abundance, we found that gamebirds contributed a small percentage of buzzard diet during the buzzard breeding season and that their provision did not influence between‐nest variation in buzzard productivity. We therefore conclude that associations between buzzards and free‐roaming gamebirds are unlikely to be a consequence of the increased availability of gamebirds as prey during the buzzard breeding season. Instead, we suggest they occur either as a function of the habitat and predator management associated with shooting management, leading to higher densities of alternative prey, or as a consequence of the availability of gamebirds as prey and/or carrion during the shooting season. In regard to the latter, we highlight the value of future research into the functional response of buzzards to gamebird availability, outside of the breeding season, on land where gamebird releases take place. Taken together, our findings suggest that the interactions between gamebird releases, and associated practices of predator control and shooting itself, require better understanding to more effectively intervene in any one aspect of this complex social‐ecological system.

## CONFLICT OF INTEREST

The authors declare no conflicts of interests.

## AUTHOR CONTRIBUTIONS

**George Swan:** Conceptualization (equal); Data curation (lead); Formal analysis (lead); Investigation (lead); Methodology (lead); Project administration (equal); Resources (equal); Validation (lead); Visualization (lead); Writing – original draft (lead); Writing – review & editing (equal). **Stuart Bearhop:** Conceptualization (equal); Formal analysis (supporting); Investigation (supporting); Methodology (supporting); Project administration (equal); Supervision (equal); Writing – review & editing (equal). **Stephen Mark Redpath:** Conceptualization (equal); Supervision (equal); Writing – review & editing (equal). **Matthew J Silk:** Formal analysis (supporting); Validation (supporting); Writing – review & editing (supporting). **Daniel Padfield:** Formal analysis (supporting); Validation (supporting). **Cecily Goodwin:** Formal analysis (supporting); Validation (supporting); Writing – review & editing (supporting). **Robbie A McDonald:** Conceptualization (equal); Funding acquisition (lead); Investigation (supporting); Methodology (supporting); Project administration (equal); Resources (equal); Supervision (equal); Writing – original draft (supporting); Writing – review & editing (equal).

## Supporting information

Supplementary Material

## Data Availability

The data and code underpinning this study are available at Dryad data repository https://doi.org/10.5061/dryad.kkwh70s6w.
