# Supplementary material for: Associations between abundances of free‐roaming gamebirds and common buzzards Buteo buteo are not driven by consumption of gamebirds in the buzzard breeding season
Source: Ecol Evol. 2022 May 3;12(5):e8877. doi: 10.1002/ece3.8877 (PMC9064828; doi:10.1002/ece3.8877)
Supplement: Supplementary file 1 — Supplementary Material [file ECE3-12-e8877-s001.docx]

Associations between abundances of free-roaming gamebirds and common buzzards *Buteo buteo* are not driven by consumption of gamebirds in the buzzard breeding season

George J F Swan, Stuart Bearhop, Stephen M Redpath, Matthew J Silk, Daniel Padfield, Cecily E.D. Goodwin & Robbie A McDonald*

*Correspondence: Robbie McDonald. E-mail [r.mcdonald@exeter.ac.uk](mailto:r.mcdonald@exeter.ac.uk)

**Appendices**

**Appendix Table S1.** Variation in habitat composition of the three sites used to study common buzzard behaviour and breeding in Cornwall, UK. Habitat assessed using the UK Land Cover Map, at 25m x 25m resolution, from the year of data collection (2015).

| Site | Improved grassland | Arable & horticulture | Broadleaved woodland | Coniferous woodland | Suburban |
| --- | --- | --- | --- | --- | --- |
| A | 38.6% | 46.6% | 12.6% | 0.0% | 1.0% |
| B | 41.9% | 11.8% | 40.3% | 4.9% | 1.1% |
| C | 36.4% | 44.8% | 16.5% | 1.2% | 1.0% |

**Appendix Figure S1**. Images from camera footage demonstrating the identification of a free-roaming pheasant (A) and a released pheasant (B) from presence of intact primary feathers or clipped primary feathers, respectively.


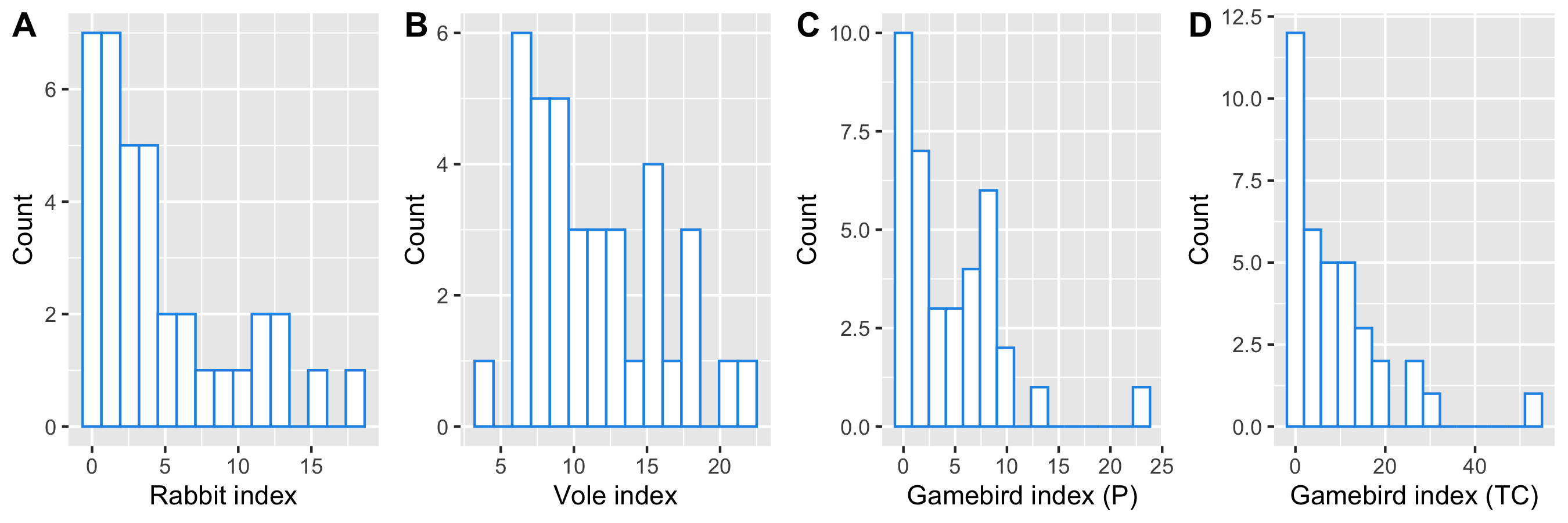


**Appendix Figure S2**. Frequency histogram plots for the relative prey abundance indices for (A) rabbits, (B) field voles and gamebirds (C = presence, D = total count) in 37 common buzzard territories in Cornwall, UK.
